# Exploring underlying factors associated with effective coverage of diabetes care in Iran: a systems thinking approach

**DOI:** 10.1186/s12913-026-15160-3

**Published:** 2026-07-24

**Authors:** Fatemeh Ehteshami, Fabrizio Tediosi, Günther Fink, Daniel Cobos Muñoz

**Affiliations:** 1 Swiss Tropical and Public Health Institute (Swiss TPH), Allschwil, Switzerland; 2 University of Basel, Basel, Switzerland; 3 University of Milan, Milan, Italy

**Keywords:** Diabetes, Effective coverage, Systems thinking, Causal loop diagram, Primary healthcare

## Abstract

**Background:**

The prevalence of diabetes has been increasing in Iran; yet, effective glycemic control remains suboptimal. This study aimed to identify the underlying root causes that influence the effective coverage of diabetes care in Isfahan Province, Iran.

**Methods:**

We conducted a qualitative study based on face-to-face semi-structured interviews with twenty-nine participants, including fourteen health workers and fifteen patients with diabetes within primary healthcare (PHC) facilities in Isfahan Province, Iran. We conducted a thematic analysis and then mapped causal relationships through a causal loop diagram and characterized system archetypes, such as limits to growth and shifting the burden archetypes.

**Results:**

We identified four themes, namely, integrated, accessible, team-based, and continuous care capacities for diabetes management, along with their corresponding facilitators and barriers. Perceived facilitators such as the implementation of standard clinical guidelines like the IraPEN, the availability of electronic information systems such as the Integrated Primary Health Care System, known as SIB, the supply of essential medications and medical equipment, basic health insurance coverage, and screening, detection, and treatment adherence by healthcare workers, all improve the coverage of diabetes care. In contrast, perceived barriers such as weak infrastructure, poor coordination across healthcare providers and between levels of care, pharmaceutical expenditures, healthcare worker shortages, task diversification and workload, time constraints of healthcare workers, and inadequate patient engagement limit effective coverage.

**Conclusions:**

Despite efforts to integrate diabetes care into the PHC context and the expansion of basic health insurance coverage, underlying constraints such as fragmented care delivery, limited accessibility, and insufficient team-based and poor continuous care continue to impede the effective coverage of diabetes care in Iran. Our findings suggest that strengthening infrastructure for integrated care within the PHC setting, enhancing financial protection for patients, particularly those with complications, recruiting adequately trained healthcare workers for diabetes care, and promoting patient engagement are essential for providing effective diabetes management.

**Supplementary Information:**

The online version contains supplementary material available at https://doi.org/10.1186/s12913-026-15160-3.

## Introduction

The burden of diabetes is substantial, affecting 11.11% of the global population (589 million adults) in 2024, and is expected to reach 12.96% (853 million adults) by 2050 [1]. The Middle East and North Africa (MENA) region is projected to experience a 92% increase in diabetic cases from 84.7 million in 2024 to 162.6 million in 2050. Iran ranks among the top five countries in this region in terms of the number of individuals affected by diabetes, with an estimated 5.5 million patients in 2024, and an expected increase to 8.2 million cases in 2050 [2]. The prevalence of prediabetes and diabetes increased from 18.11% to 10.58% in 2016 to 24.81% and 14.15% in 2021 [3]. While the estimated prevalence of diabetes is increasing, a nationally representative survey revealed that glycemic control is achieved in only 36.7% of cases, with 73.3% of individuals diagnosed and 65% receiving treatment coverage in 2021 [4]. Further evidence [3–8].

The complex nature of diabetes, with the presence of comorbidities such as hypertension and cardiovascular disease complications [9, 10], particularly within a complex healthcare system, places notable pressure on both health systems and patients. Among diabetes-related complications, the prevalence of cardiovascular disease in patients with type 2 diabetes in Iran was 37.4% between 2001 and 2019 [11]. Cardiovascular disease remains the leading cause of mortality and morbidity in Iran, accounting for 46% of all deaths [12].

In response to this growing epidemic condition, the World Health Organization(WHO) launched the Global Diabetes Compact (GDC) in 2021, structured around three key components, including access to insulin and medical technologies, strengthening health systems’ resilience, and engaging individuals affected by diabetes and co-create solutions [13]. The GDC seeks to advance the implementation of the WHO Global Action Plan for the prevention and control of noncommunicable diseases (NCDs) 2013–2020 [14], which prioritizes early diagnosis of diabetes, equitable access to essential insulin and technologies, enhancing healthcare providers’ skills and capacity, while emphasizing continuous quality improvement, information systems and electronic medical records, and development of diabetes registries within the health systems to support both patient care and population-level monitoring [15]. The WHO also identified the WHO package of essential noncommunicable disease (PEN) interventions for primary health care in low-income settings (WHO PEN) [16, 17] and WHO best buys [18] as cost-effective, evidence-based interventions, especially relevant in resource-constrained settings. Nevertheless, in many countries, particularly in low and middle-income countries, treatment for diabetes has not increased adequately in comparison with rising prevalence, resulting in a disproportionate burden and untreated diabetes [19].

In Iran, the Ministry of Health and Medical Education (MOHME) in 2013 initiated a national strategy for the prevention and control of NCDs and their risk factors by establishing a national committee for NCDs and launching a national action plan for the prevention and control of NCDs and the related risk factors from 2015 to 2025 [20]. In addition, the WHO PEN [16] was adapted to IraPEN and integrated into Iran’s primary health care (PHC) network across all provinces [21]. However, despite initiatives for NCDs management in Iran, the effective diabetes coverage in Iran remains unsatisfactory, particularly in underserved settings, as a cross-sectional study highlighted that financial, educational, social, and healthcare access needs, such as health insurance coverage, medication support, and access to monitoring tools, are largely unmet for type 2 diabetes [22]. Additionally, evidence outlines that despite the availability of healthcare services and routine check-ups for patients with type 2 diabetes in Iran, gaps in healthcare service quality persist. Rural populations face greater disparities in Iran [23].

The pursuit of effective coverage for diabetes takes place within a complex adaptive system in which various individuals, actors, entities, and behaviors are involved. An optimal approach to unpacking such complexity is to adopt a systems thinking lens. The key characteristics of the systems thinking approach include understanding interconnections, identifying dynamic behavior and feedback loops, and recognizing leverage points through tools such as causal loop diagrams and system archetypes [24].

Therefore, this qualitative study, through a systems thinking approach, aims to seek facilitators and barriers, drawing on the perceptions and experiences of both patients and health workers to identify the root causes of suboptimal effective coverage of diabetes care in Isfahan Province, Iran.

## Methods

### Study design

This qualitative study, conducted in Iran in 2023, involved two groups of participants, including health workers and patients with diabetes. We conducted a thematic analysis, and our findings were visualized through a causal loop diagram to illustrate the underlying cause-and-effect relationships. The qualitative study was useful in this study to address the research questions, as this approach provides detailed insights into a specific phenomenon through examining contents, dynamics, and processes via comprehensive and rich data [25]. Qualitative research paves the way for deriving and interpreting underlying patterns grounded in a systems thinking lens. Our study followed the guidelines of the Consolidated Criteria for Reporting Qualitative Research (COREQ), guided by a checklist to facilitate the description of the methods, data analysis, and results [26].

### Study setting

In Iran, the MOHME is the primary provider of healthcare services in Iran through a nationwide of provincial medical universities. Primary care within the PHC network is publicly funded and provided by the government [27, 28]. Secondary and tertiary care are provided by various entities; however, one of the main public providers other than the MOHME is the Social Security Organization (SSO), which is also one of the major public purchasers of healthcare services. The SSO operates under the supervision of the Ministry of Cooperatives, Labor, and Social Welfare, providing both direct and indirect outpatient and inpatient healthcare services at the secondary and tertiary levels [29]. Under the direct approach, services and medical products are provided and delivered through clinics and hospitals owned by SSO, which are located primarily in urban areas. The coverage is delivered directly to insured individuals through 67 accredited hospitals and 270 clinics, such as diabetes clinics, across the country. Alternatively, the indirect approach involves purchasing healthcare services and goods from public and private providers. SSO insurance provided coverage for approximately 42% of the population in 2017 [28, 30, 31]. In addition, the Iran Health Insurance Organization (IHIO) is another social health insurance scheme that provides basic health insurance coverage through an indirect approach. The IHIO is structured by five sub-funds that serve governmental employees, the self-employed, rural populations with fewer than 20,000 inhabitants, previously uninsured individuals, and groups with particular social protection needs [32]. The delivery of healthcare services for NCDs, such as diabetes, has been influenced by various reforms, strategies, and policies. The family medicine program and integrated healthcare referral system in rural areas in 2005 [33] and the health transformation plan in 2014 [30] were two significant reforms in the health system of Iran.

At the provincial level in Iran, the University of Medical Sciences is responsible for public health and the provision of healthcare services, in addition to medical education. All universities of medical sciences across all provinces follow the same PHC structure under the national supervision of the MOHME [34]. Therefore, we focused on Isfahan Province, one of the largest provinces in Iran, with a total population of 4,791,905 and 2,744,053 individuals over the age of 30 [35]. The prevalence of diabetes in this province was 11,59% in 2021 [35], yet only 35,43% of the population was under control, including 30,05% in rural areas and 36,08% in urban areas [36]. Our focus was on the PHC network in both urban and rural areas, where we approached individuals at village health houses, rural health centers, urban health posts, and urban health centers in a district under the supervision of Isfahan University of Medical Sciences.

### Study participants and recruitment

The study was conducted through one-on-one semi-structured interviews with health workers and diabetic patients. The health workers interviewed included general practitioners (GPs) such as family physicians in rural areas and primary care physicians in urban areas, community health workers (CHWs), health technicians in urban areas, and district health officials. The first author (FE) personally interviewed all the participants. The interviews took place in health facilities, as well as in the workplaces of health officials. We invited the participants based on their convenience and their available time to conduct the interviews. District health officials from the provincial health district and the district health center were invited directly by FE. Health officials subsequently informed the healthcare workers of the PHC facilities through formal letters and telephone calls about the aim of this research. FE personally and directly attended the PHC facilities and invited healthcare workers. They agreed to participate in the interview based on their availability and interest. The selected health workers had specialized knowledge about healthcare services for cardiometabolic risk factors such as diabetes. We excluded health workers who were not directly engaged in healthcare services for diabetes. Thus, both purposive and snowball sampling techniques were used to identify health workers. Snowball sampling refers to a non-probability sampling method in which a researcher starts with a small group of known individuals and expands the sample by requesting the initial participants to refer other people who meet the study’s criteria [37]. The purposive sampling technique, also known as judgment sampling, involves the deliberate choice and selection of participants who are information-rich cases on the research topic [38]. In addition, healthcare workers at health facilities assisted us in inviting diabetic patients. We only included patients with type 2 diabetes with/or without comorbidities such as hypertension, dyslipidemia, and cardiovascular disease. Patients with other types of diabetes, such as gestational and type 1 diabetes, were excluded. FE interviewed patients who were both available and interested. The sample size for the interviews was determined by saturation, i.e., participant recruitment ceased when no new relevant information emerged concerning the research questions [38] for each group of participants.

### Data collection

We conducted semi-structured interviews with the participants. The interviews were conducted from September 2023 to November 2023.

The interview guides were developed by the research team based on the research objectives (Appendix 1). The interview guides were piloted with two participants from each group to ensure clarity and relevance. The interviews included open-ended questions that allowed both groups of participants to freely share their additional perceptions and expectations for improving the management of diabetes. The interviews with the health workers began with the question “*How*,* where*,* and by whom are the diabetic patients screened*,* diagnosed*,* and treated?*”. This question enabled the research team to capture healthcare service delivery along the cascade of care for diabetes patients. The interviewer also encouraged health workers to express barriers that hinder effective healthcare service delivery through questions such as “*Can you explain any barriers*,* delays along the cascade of care?*”. The interviews then continued with questions such as “*What factors contribute to the loss of diabetic cases at each stage of the cascade of care? Why?*”. These questions enabled the research team to identify the root causes and relationships influencing the effective coverage of diabetes.

The interviewer also approached patients to identify their perceptions and experiences regarding the healthcare services of diabetes; thus, the questions began with “*how*,* where*,* and by whom was your disease diagnosed?*” The interview proceeded with the question, “*Since your diagnosis*,* where and how have you been receiving regular follow-up and treatment?*”. Subsequently, it continued with questions such as “*How do you describe your experience with the delivery of healthcare services?* “*What challenges and difficulties*,* if any*,* have you faced when receiving healthcare services for your condition?*”. These questions allowed the research team to understand the causal links of poor management of diabetes. Moreover, both groups of participants were free to share their additional perceptions as well as expectations for improving the management of diabetes.

The interview guide, topic, and research aim were discussed before the interviews started. Interviews were conducted only once. All the interviews were conducted in Persian, and the average interview time was 21 min for health workers (minimum 9 min and maximum 54 min) and 11 min for patients (minimum 4 min and maximum 23 min). The interviews were carried out via voice recording and note-taking. Five participants refused to be tape-recorded, and FE took notes during the interviews. Upon request, study participants were given an anonymized copy of their transcript. The participants were informed that the research sought to understand their knowledge, experience, and perceptions of diabetes management in Isfahan Province, Iran.

### Ethical aspect of the research

Ethical approval was obtained from the Ethics Committee of Northwest and Central Switzerland (EKNZ) (Statement ID: AO_2022-00069) and Tehran University of Medical Sciences (Approval ID: IR.TUMS.MEDICINE.RER.1401.432). The study was performed in accordance with the guidelines of the Declaration of Helsinki. Participation in the interview was voluntary, and participants were free to withdraw at any stage. The interviews were conducted after informed consent was obtained from all participants. During transcription, names and other details that would lead to a direct link to an individual were anonymized.

### Data analysis

We conducted an inductive thematic analysis using the framework method [39]. Framework Method is a flexible and adaptable tool that can be employed within multiple qualitative approaches. The tool itself carries no allegiance and fixed orientation to either inductive or deductive thematic analysis, where the extent to which it leans in the inductive-deductive continuum is shaped by the research question [39]. We therefore adopted the following stages:

First, the interviews were transcribed verbatim. The transcripts were not returned to the participants for comments or corrections. We translated the transcripts from Persian to English. Second, FE reviewed the transcripts to grasp an overall content, while engaged in a more familiarization process by repeatedly reading each transcript and listening to the audio-recorded interviews (familiarization). To initiate inductive coding, FE underlined noteworthy segments of text and assigned short descriptive labels and codes, alongside recorded reflective notes such as questions, explanations, and interpretations (coding). Subsequently, FE and DC discussed and refined these preliminary codes, clustered them into facilitators and barriers codes, and agreed on an initial analytical framework. Using an initial analytical framework, FE reviewed the transcripts several times carefully and coded all transcripts, while also noting emergent codes and revising the framework accordingly. Later, FE and DC grouped related codes into categories (developing and applying the analytical framework). Next, the data were organized into a framework matrix, with rows representing participants and columns representing codes, grouped into categories (charting data into the framework matrix). The themes were informed by the concepts emerging inductively from the dataset. In addition, the developed framework matrix, where participants, codes, and categories were summarized, enabled us to identify and map the underlying causal relationships (Interpreting the data). We used MAXQDA 24 to facilitate data management.

We applied causal loop diagrams using Vensim PLE x64 software to facilitate our visualizations. Causal loop diagrams, which are referred to as mental models, provide insights by mapping dynamics, interdependencies, and the underlying causal linkages [40, 41]. A causal loop diagram contains variables, causal links with positive (+) or negative polarities (-), feedback loops, and delays. In a causal loop diagram, two system elements (variables) are connected by arrows. A positive polarity indicates a relationship in which an increase in one variable influences the other variable in a similar way. Conversely, a negative polarity shows that a decrease or an increase in one variable produces an opposite effect on the other variable. Feedback loops, as key features of causal loop diagrams, represent the drivers of system behavior, such as reinforcing and balancing loops [40, 42]. In addition, identifiable patterns of behaviors and interactions recur within complex systems across various contexts. These recurring patterns are known as systems archetypes, which are generic, simplified, and powerful causal loop diagrams that capture how feedback loops, such as balancing loops and reinforcing loops, interact to lead to particular patterns over time. Each archetype is characterized by a distinct storyline [43–45]. Thus, a set of systems archetypes may be derived from the key causal relationships.

We adhered to the guidelines from Lincoln and Guba [46, 47] to ensure the study’s trustworthiness. To ensure reflexivity, FE engaged in regular self-reflection to examine personal assumptions and preconceptions about the research topic. In particular, FE maintained a reflexive journal throughout the data analysis process. In addition, FE engaged in peer debriefing with DC, who challenged the first author with critical questions to uncover potential biases, perspectives, and assumptions in the interpretation of findings, a process known as peer debriefing. Moreover, the existing literature and documents allowed us to gain a deeper understanding of diabetes care in Iran and to strengthen the validity of the findings. The first author retrieved and reviewed related existing published evidence in Iran, in English and Persian, and also relevant national and provincial documents in Persian, such as laws, regulations, policies, plans, and reports. In addition, the district health officials shared with the first author relevant published documents, such as guidelines, policies, and strategies in Persian. Neither health workers nor patients were invited to provide feedback on the findings.

## Results

### Sample characteristics

We conducted interviews with fourteen health workers and fifteen patients with diabetes. The health workers included three district-level health officials, six CHWs and health technicians, and five GPs. CHWs and GPs in rural areas were employed under the family physician program. Among the patients, three had only diabetes, and twelve had at least one cardiovascular risk factor, such as hypertension and dyslipidemia, other than diabetes (Table 1).

**Table 1:** Demographic characteristics of the participants

Characteristics of participants	Number	%
**Health workers**		
Total	14	100
GP	5	36%
CHW and health technician	6	43%
Official	3	21%
**Gender**		
Female	10	71%
Male	4	29%
**Age**		
30–40	4	29%
41–50	9	64%
51–60	1	7%
**Diabetes patients**		
Total	15	100
**Gender**		
Female	10	67%
Male	5	33%
**Age**		
30–40	1	7%
41–50	0	0%
51–60	9	60%
Over 61	5	33%

### Drivers influencing the effective coverage of diabetes

**Fig. 1:**
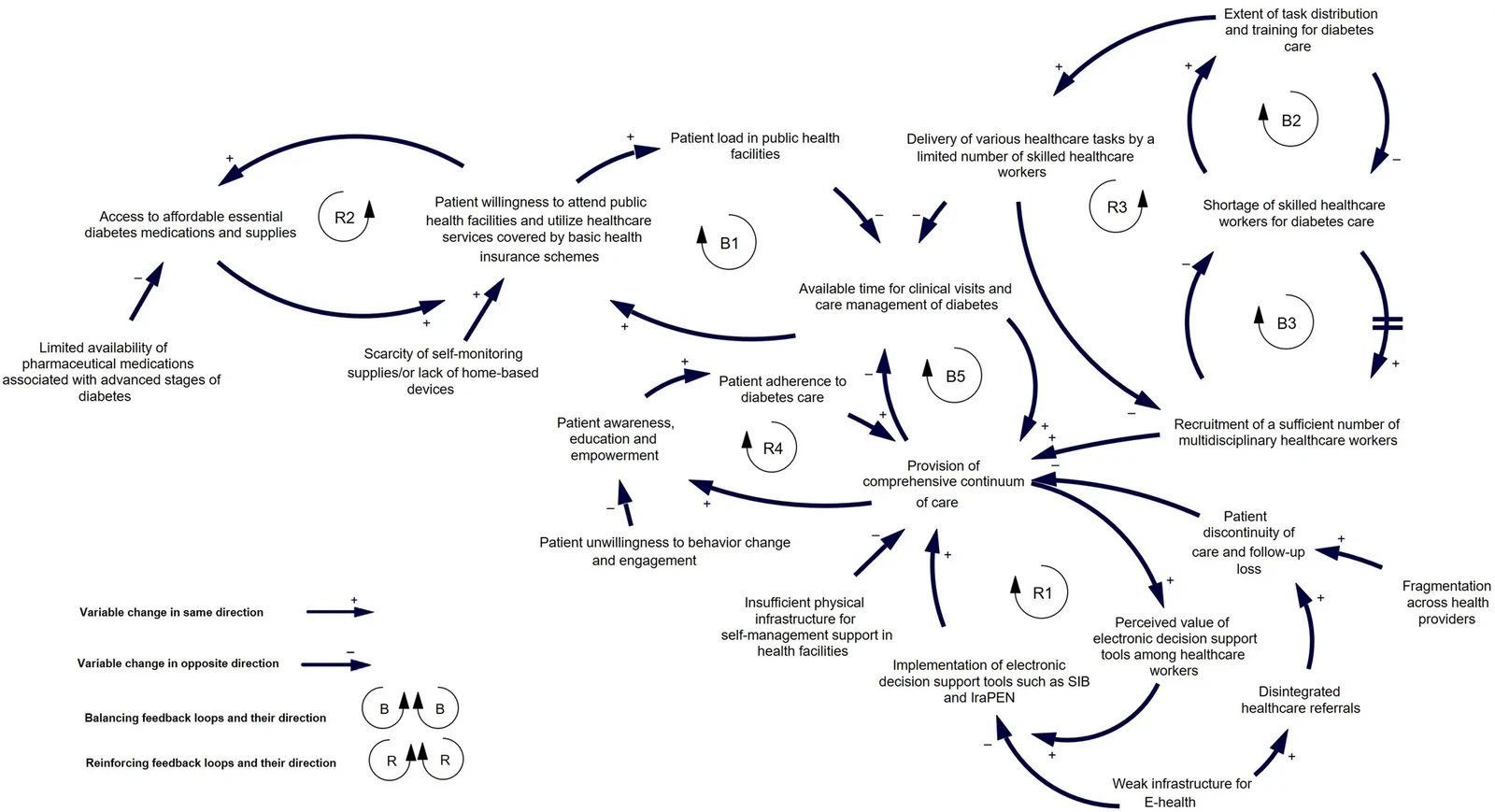
Causal loop diagram of diabetes management in Isfahan Province, Iran, highlighting the key causal relationships

We identified twenty-two facilitator and barrier codes, clustered them into eight categories, which were grouped into four overarching themes, including integrated, accessible, team-based, and continuous healthcare service capacities within the PHC context in Isfahan Province (Table 2). In addition, the causal relationships derived from the analysis are shown in Fig. 1.

**Table 2:** Identified facilitators and barriers affecting the effective management of diabetes within the PHC context

Themes	Categories	Facilitator codes	Barrier codes
Integrated healthcare services	NCD care integration into PHC through electronic health information systems and decision support tools	Implementation of adapted WHO PEN guidelines, known as IraPEN	Weak infrastructure for the electronic health information system
		Implementation of the Integrated Primary Health Care System, known as SIB (Persian acronym سیب)	
	Care coordination		Time-consuming process of the SIB
			E-referral deficiencies
			Fragmented care within the care levels and across healthcare providers
Accessible healthcare services	Affordability and coverage	Basic health insurance coverage for medical access	Pharmaceutical expenses for diabetes, particularly complicated stages
	Medications and supplies availability	A comprehensive supply of essential medical equipment and affordable tests in primary care	Insulin rationing
			Limited access to self-monitoring supplies, such as strips for the blood glucose meters
Team-based healthcare services	Healthcare workers’ availability, job demand, and responsibilities of the care team	Task distribution and collaboration through the SIB	Shortage of trained health workers in diabetes care
			Task diversification and workload
			Non-clinical task overload
			Insufficient motivation due to high workload
	Empowerment and competency of the care team	Regular training of health providers organized by the medical university	Inadequate knowledge in managing diabetes care, lack of confidence
Continuous healthcare service	Adherence to treatment	Regular screening, detection, follow-up, and treatment by CHWs and health technicians based on IraPEN guidelines, and SIB reminders	Inadequate patient engagement and willingness to behavior change
	Self-management support and education		Time constraints on clinical visits and case management for diabetes
			Poor infrastructure for education and self-management support, such as physical spaces for group education

#### Theme 1: Integrated healthcare services

This theme was generated particularly from the perceptions of the health workers. This theme comprises two categories, including NCD care integration into the PHC and care coordination. Facilitators such as the implementation of adapted WHO PEN guidelines, known as IraPEN, and the implementation of the Integrated Primary Health Care System, known as SIB (Persian acronym سیب), improve the integration of NCD care within the PHC. The SIB also enhances care coordination. However, barriers such as weak infrastructure for electronic health information systems, e-referral deficiencies, time-consuming process of the SIB undermine the integration of NCD care and negatively affect the care coordination. In addition, fragmented care within the care levels and across healthcare providers weakens the care coordination.

The integration of NCD care into primary care through the implementation of IraPEN guidelines and an electronic health information system, known as SIB, was perceived by health workers as a means of improving diabetes management. An official reported, “*We adapted the national IraPEN to the context of the province. By the end of 2015*,* the SIB was operational in all primary health facilities*,* and all risk assessments related to IraPEN guidelines were imported into the SIB*” (H14). A family physician explained, “*The SIB serves as a helpful tool to remind us to schedule and attend follow-up appointments for patients*” (H5). Similarly, a GP in urban primary care stated, “*The strength of this approach [IraPEN] is that it provides a clear decision-support guideline for the treatment and adherence process. We follow its instructions*” (H3). However, some participants expressed concerns that the SIB is time-consuming due to the weak infrastructure for this system. One of the CHWs said, “*We face challenges related to infrastructure*,* such as internet connections*,* because our primary work depends on electronic health. When it is weak and unstable*,* it negatively impacts our performance*,* our satisfaction*,* and our motivation. Internet connection is really unstable in health houses*” (H9).

In addition, there is no integration between PHC facilities and SSO clinics. A GP stated, “*We follow up patients with SSO health insurance who would attend those clinics [SSO-clinics]; however*,* we are two different systems and do not have any collaboration and connection*” (H10). Thus, healthcare workers highlighted the necessity of establishing a specialized clinic for diabetes in primary care in the district to address diabetic patients’ needs and ensure comprehensive care. A family physician explained, “*The diabetes clinic of the district was terminated 6 years ago. Since then*,* there have been no specialized clinics in the primary care of the district*,* except for those in the SSO clinics. The specialized clinics are commonly found in the private sector*” (H8).”Another GP also stated, “*We previously had such clinics [diabetes clinic] in the district*,* and we knew patients were under follow-up and control of trained physicians and nurses*,* and we referred them to the clinic. It was in our system [under the supervision of the district health office]*,* and we were able to track the patients. Education of patients on diabetes is time-consuming*,* and I am responsible for many other patients. I believe there is a need for a dedicated center for diabetes care in each district*” (H3).

A further perceived barrier is the ineffective integration of the referral system between primary and secondary care within the PHC networks. Electronic health referral (E-referral) also remains in an early stage of development, and its implementation has faced several structural and operational deficiencies. A respondent explained, “*It is not possible to observe the specialist treatment. E-referral is a new approach*,* and the infrastructure is weak. The district hospital does not admit patients without an appointment from my side [primary care physician]*,* but it is challenging to use this platform for scheduling appointments*” (H3).

**Fig. 2:**
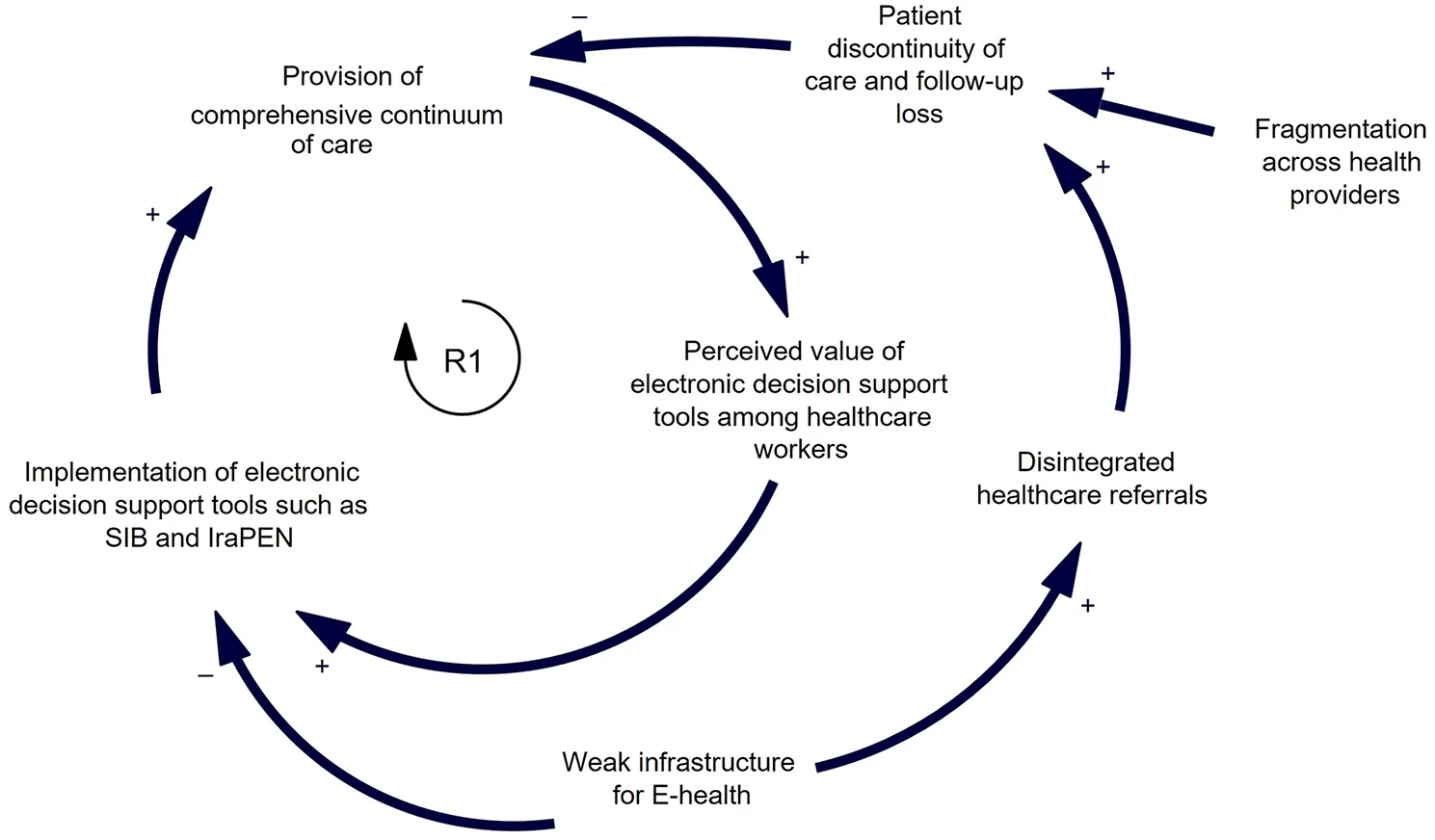
A reinforcing loop (R) and the causal relationships of integrated healthcare services for diabetes care in Isfahan Province, Iran

Figure 2 shows a reinforcing loop in which the integration of SIB and IraPEN enhances the provision of a comprehensive continuum of care, including detection, diagnosis, treatment of diabetes, and self-management support, which contributes to perceived value among healthcare workers, thereby encouraging adoption of these supportive tools (R1). Weak e-health infrastructure, such as undeserved internet connectivity, limits the effective implementation of SIB and IraPEN. Moreover, deficiencies in e-health infrastructure negatively impact the integration of healthcare referrals, leading to patient discontinuity and follow-up loss, as healthcare referral back is inadequate, and primary care physicians are not able to track and monitor patients’ treatment at higher levels of care. We also found that fragmentation across health providers contributes to patient care discontinuity and follow-up loss, as well as poor patient tracking, as health providers, such as PHC facilities and SSO clinics, are not integrated in a collaborative and coordinated way.

#### Theme 2: Accessible healthcare services

Through our analysis, this theme was identified through two categories, including affordability and coverage, medications and supplies availability. Participants highlighted facilitators such as basic health insurance coverage for medical access, a comprehensive supply of essential medical equipment, and affordable tests in primary care, alongside barriers, such as pharmaceutical expenses for diabetes, particularly complicated stages, insulin rationing, and limited access to self-monitoring supplies, such as strips for the blood glucose meters.

Health workers perceived the availability and large-scale distribution of essential medical equipment at primary health facilities provided by Isfahan Medical University as a key strength. An official stated, “*We have supplied LipidPro devices to [primary care] facilities in Isfahan. They are rapid tests that enhance screening*,* detection*,* and regular follow-ups of patients with prediabetes and diabetes. When a facility lacks the device*,* patients are referred to a laboratory free of charge for the first time and at a reduced cost for subsequent tests*” (H14).

Limited access to insulin based on health insurance coverage was perceived as a challenge. “*The issue of access to insulin involves rationing and a fixed allocation of insulin pens. Under the health insurance system*,* the quantity of insulin prescribed is determined based on the patient’s needs*,* as assessed by a specialist or board-certified physician. Since specialists are responsible for prescribing insulin*,* patients are often required to visit them repeatedly*” (H3). A patient said, *“… the specialist should prescribe it [insulin]*,* after which I go to the pharmacy; if it is unavailable*,* resulting in the need to go back and forth to pharmacies. I also cannot visit a specialist in private clinics because it is expensive for me*” (P14). Moreover, many patients complained about the scarcity of test strips for blood glucose meters for self-monitoring. “*I have a device at home*,* but I struggle to find the strips and need to search a lot*,* so I would like to visit the nearest health facility for my check-ups*” (P8). The availability of essential supplies at PHC facilities promotes patients’ willingness to utilize healthcare services and adhere to routine check-ups at nearby facilities. A patient stated, “*I do not have a device at home*,* but since the health house is close to us*,* and Behvarz is helpful and competent*,* I’d like to have my regular check-ups here*” (P12).

Financial strain related to the management of progressed diabetes with complications also presents a further barrier. A family physician said, “*I usually prescribe metformin*,*… but the range of available medications has significantly expanded. In my opinion*,* the key challenge might be the price of the diabetes medications*” (H5). A patient with diabetes and cardiovascular disease also stated, *“I buy my medicine out of pocket; they have been so expensive*” (P1).

Health insurance plays a crucial role in providing access to affordable essential diabetes medications. For instance, antidiabetic drugs such as metformin and combination drugs such as Zipmet are covered either free of charge or subject to a low deductible for patients with SSO and IHIO coverage. A healthcare worker stated, “*Many patients would go to SSO clinics … medications are affordable*” (H10). Another healthcare worker added, “*We [PHC team] have more hypertensive patients than diabetic patients. Perhaps*,* their medicines [diabetic patients] are expensive; they would attend SSO clinics. Most patients go to those clinics and perform their lab tests and bring them to us*,* and we record them in our system [SIB]*” (H3). In addition, patients with rural health insurance under IHIO coverage prefer visiting district health hospitals, as their expenses are more affordable. *“I do not have SSO insurance. I have rural health insurance coverage. Therefore*,* I go to the hospital to see a specialist and receive my heart medication. The hospital is crowded*” (P14). However, many patients emphasized that public diabetes clinics, such as SSO clinics, are crowded. A patient stated, “*My physician booked an appointment for me to see a specialist at the district hospital*,* but it was too crowded*,* so I went to a private clinic*” (P8).

Moreover, not all medications are fully included under the coverage of SSO or rural health insurance provided by IHIO, as health insurance policies are often limited to specific dosages and certain categories of medications. A physician added, “*If we change the dosage of a medication*,* it may no longer be covered by SSO insurance; so*,* we should prescribe the medication with the specified dosage that is covered by the health insurance*”(H10). One patient said, “*There is no cost for my hypertensive medication*,* but my heart medication is not covered [by health insurance]*” (P14). Another patient said, “*The expenses are hard*,* but what I can do*,* medicines are expensive and I should buy them every month*” (P15).

**Fig. 3:**
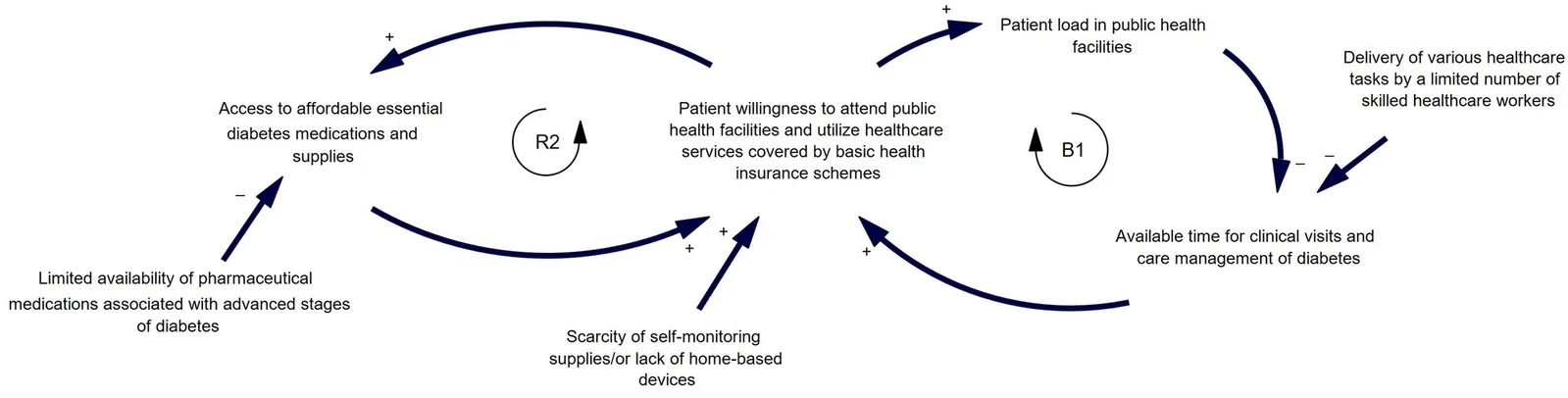
Limits to growth archetype of accessible healthcare services for diabetes care in Isfahan Province, Iran

In Fig. 3, a “limits to growth” archetype is shown, in which patients’ willingness to utilize healthcare services in public healthcare facilities improves access to affordable essential medications and supplies, thereby reinforcing their willingness to seek care in public health facilities (R2). However, limited access to pharmaceutical medications associated with advanced stages of diabetes in public health facilities negatively influences access to affordable diabetes medications. We also found that a high patient load in public health facilities decreases the time for sufficient care management of patients, which in turn reduces patients’ willingness to utilize public healthcare services (B1). The scarcity of self-management supplies, such as strips for blood glucose meters or a lack of home-based monitoring devices, leads patients to seek care at public health facilities.

#### Theme 3: Team-based healthcare services

This theme emerged from the perception and experience of both health workers and patients. Healthcare workers’ availability, job demand and responsibilities, and empowerment and competency of the care team were identified as the categories. Codes within this theme highlighted both facilitators such as task distribution and collaboration through the SIB and regular training of health providers organized by the medical university as well as barriers, such as shortage of trained health workers in diabetes care, task diversification and workload, non-clinical task overload, insufficient motivation due to high workload, and inadequate knowledge in managing diabetes care, and lack of confidence.

The training of healthcare workers was considered essential, and the recent training initiative by the medical university was perceived positively. Despite this, many healthcare workers asserted that diabetes care requires specialized knowledge. “*Previous training sessions focused on IraPEN guidelines. Furthermore*,* the medical university organized a diabetes course*,* but as a primary care physician*,* I would not prescribe insulin*” (H3).

Some participants perceived that the SIB allows primary healthcare teams to collaborate, assign, and distribute responsibilities such as treatment adherence and follow-up more efficiently. A health worker stated, “*We distribute and allocate tasks and responsibilities within our health facility by assigning a specific number of patients to each personnel*,* using a blocking system in SIB in which patients are categorized based on their demographic characteristics. On average*,* we follow up with approximately 10 to 15 individuals with diabetes assigned to us each day*” (H4).

Many healthcare workers viewed the limited number of healthcare workers, task diversification, high workload, and non-clinical task overload as barriers to offering effective management for diabetes. An official stated, “*We have a shortage of human resources*” (H12). A health technician added, “*We have a nutritionist available two days a week. Our facility [Urban health center] has a physician every day; however*,* some villages and rural areas have only one day a week*,* which is a challenge*” (H4). A CHW also stated, “*The workload is excessive*,* with various programs targeting different groups such as children and schools*,* pregnant women and newborns*,* and infectious diseases. Some events like the week of the elderly further increase our workload*” (H6). With respect to non-clinical tasks, a respondent said, “*We dedicate our time to filling out checklists for different conditions*,* often required for quality assessment. We also have to switch between multiple E-platforms*,* which further contributes to the time burden*” (H10). In addition, task diversification and workload may influence the motivation to provide effective management of diabetes by healthcare workers. “*I monitor the health facilities and see that some personnel have time*,* but would not like to undertake the duties*,* as it is crowded and there are different tasks*” (H14).

Healthcare workers emphasized the need for specifically trained healthcare workers to be responsible for the care of diabetes patients at each health facility. “*Specialized staff should be available specifically for diabetes and cardiometabolic risk factors [at the PHC facilities]*” (H11). Furthermore, a health worker said, “*…In this facility*,* the population is approximately 17*,*000*,* and I am currently the only physician. However*,* at least two physicians are needed to adequately serve this population. For us*,* all aspects of care matter; pregnancy care is very important*,* student health is important*,* and we dedicate considerable time to NCDs as well. It is the physician’s sense of responsibility*,* but this situation is far from standard*,* in my opinion*” (H3).

**Fig. 4:**
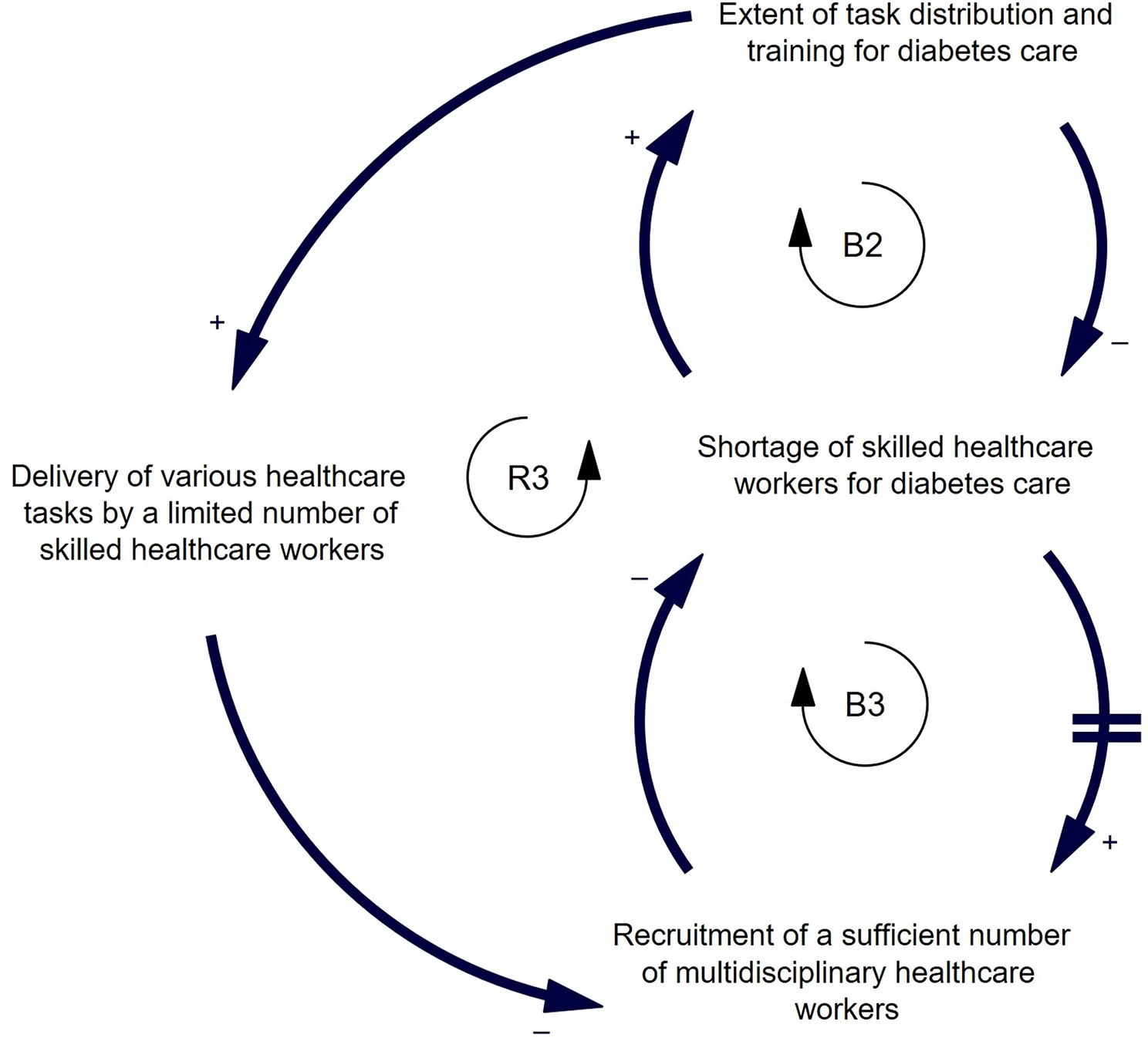
Shifting the burden archetype of team-based healthcare services for diabetes care in Isfahan Province, Iran

We developed a “shifting the burden” archetype, in which the shortage of skilled healthcare workers for diabetes care within PHC facilities increases task distribution and necessitates regular training for various conditions, such as diabetes care, serving as a short-term symptomatic solution to the shortage of healthcare workers. This increases the dependency on these solutions and delays efforts to address the underlying root causes (B2). However, these quick-fix solutions lead to the implementation of various healthcare tasks by a limited number of healthcare workers as a side effect of the quick-fix solution. This may contribute to delays in implementing long-term strategies aimed at enhancing the recruitment of trained and specialized healthcare workers for diabetes, which was perceived by participants as a fundamental solution to decrease the insufficient number of multidisciplinary, skilled healthcare workers for diabetes care (R3). This highlights the relevance of addressing the root cause of the problem to improve team-based care in the long run (B3) (Fig. 4).

#### Theme 4: Continuous healthcare services

This theme comprised of two categories, including adherence to treatment and self-management support and education, emerging from both health workers and patients. The supporting codes illustrated facilitators, e.g., regular screening, detection, follow-up, and treatment by CHWs and health technicians based on IraPEN guidelines, and SIB reminders. Barriers were also identified, including inadequate patient engagement and willingness to behavior change, time constraints on clinical visits and case management for diabetes, poor infrastructure for education and self-management support, such as physical spaces for group education.

According to IraPEN and the integration of NCD care into primary care and the implementation of SIB, regular screening, detection, follow-up, and treatment by CHWs and health technicians have improved continuous care. A CHW said, “*When the disease is diagnosed*,* based on guidelines and SIB reminders*,* we initiate monthly follow-ups*,* and physicians every three months*” (H1). Patients also noted regular follow-ups and support from CHWs serving their catchment population. “*If we miss one month of follow-up*,* they call us and check in*”. In addition, patients reported effective communication with the family medicine team, particularly in rural villages. “*The physician spends a lot of time; each patient takes 20–30 minutes*,* and we ask all our questions*” (P10).

Due to time constraints, healthcare workers, and especially GPs, often struggle to provide case management and education to diabetic patients. A respondent stated, “*I would like to spend approximately 20 minutes with my diabetic patients*,* but I have to be in charge of patients who are on the other side of the door. It makes a difference when you are concentrated on your diabetic patients*,* or you have patients with various conditions*” (H9). Some patients also reported poor communication within SSO clinics, particularly when they are crowded and waiting times are high. *“…but it is crowded*,* and I cannot talk to my physician enough*” (P13).

Patient engagement and willingness for behavior change were perceived as crucial for the effective management of diabetes. A physician said, “*Some patients are reluctant to change their lifestyle that I have been teaching in the past few years. Some diabetic patients do not care about their nutrition*” (H3). A CHW concerning patient engagement and willingness to attend group education also stated, “*We organize the group education. If we invite 50 patients*,* only about 5 individuals would attend*” (H6). A family physician believed that awareness is key to the management of diabetes. “*Now we have about 100% infant vaccination*,* mothers care about their newborns*,* which means awareness. Awareness should reach a level where individuals are fully informed about diabetes. The challenge lies in engaging patients*,* which should improve through education*” (H5). Additionally, the limited availability of resources and physical spaces for education and self-management support was perceived as a barrier to effective, continuous care. A CHW stated, “*Group education is difficult because of a lack of infrastructure… I do not have any spaces to organize that and even lack time for organizing group education*” (H9).

**Fig. 5:**
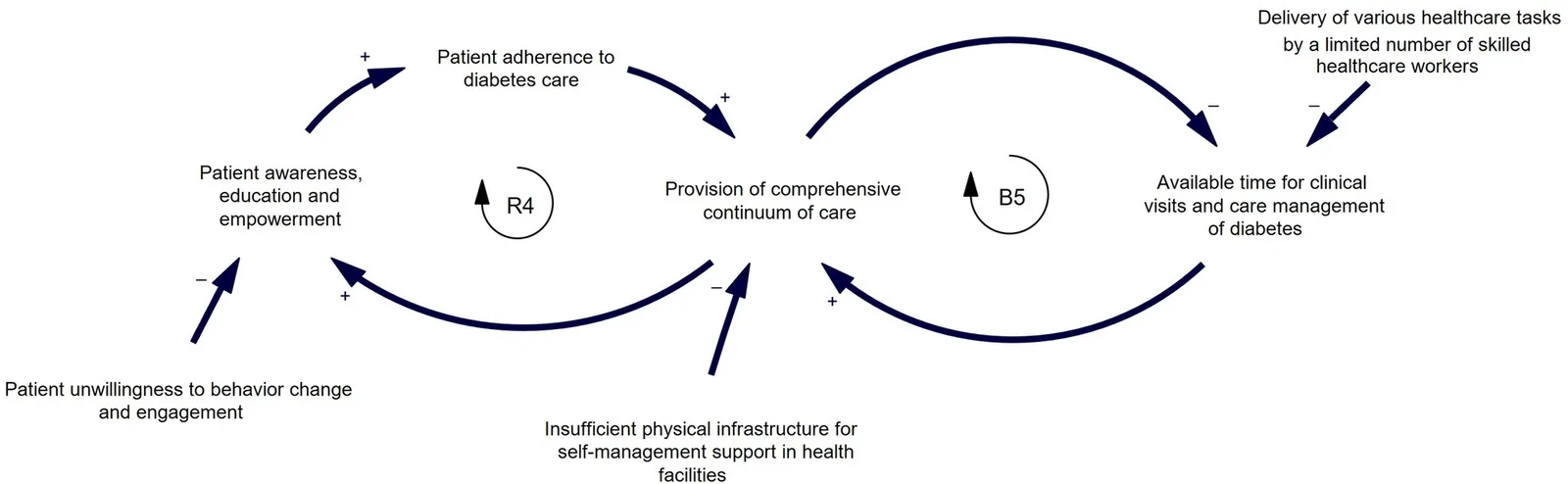
Limits to growth archetype of the comprehensive continuum of care for diabetes in Isfahan Province, Iran

A “limits to growth” archetype was developed in which the provision of a comprehensive continuum of care, such as treatment, self-management support, and education by healthcare workers, improves patient awareness and empowerment, thereby contributing to patient adherence to care (R4). However, time constraints for clinical visits and care management of diabetes patients counteract the effective delivery of the care continuum (B5). Moreover, insufficient physical infrastructure negatively influences the care provided by health workers, particularly in terms of education and self-management support. Patient unwillingness to behavior change and engagement also limits the level of patient awareness and empowerment (Fig. 5).

## Discussion

This study highlights the determinants of effective coverage of diabetes care based on the perceptions of health workers and patients within the PHC setting in Isfahan Province, Iran. We identified facilitators and barriers of four overarching themes, namely integrated, accessible, team-based, and continuous care capacities and then mapped the underlying root causes using a causal loop diagram and identified emerging systems archetypes such as limits to growth and shifting the burden archetypes.

One of the underlying themes that emerged from our analysis was integrated healthcare services, encompassing the NCD care integration in the PHC and care coordination, in which both facilitators and barriers were reflected. The integration of NCDs, such as diabetes, is facilitated by the implementation of standard clinical guidelines such as the IraPEN program. The effectiveness of the IraPEN program was assessed in a previous study, which revealed that the program modestly reduced CVD risk and associated risk factors in Iran, although further modifications may be required to improve its effectiveness [48]. Another study found that diabetes management under the IraPEN program extends to only a quarter of patients, and effective glycemic control was observed in approximately 39% of diabetic patients who had at least one HbA1c laboratory test [49].

Our findings indicate that the SIB, a significant electronic information system in the PHC context in Iran, plays a crucial role in facilitating the implementation of the IraPEN program. However, weak infrastructure, such as unstable internet connections, hinders healthcare workers’ performance and subsequently influences the effective coverage of diabetes care. A study also highlighted several challenges, such as internet disconnections during office hours and increased workload of physicians through the implementation of SIB in Iran [50]. Similarly, a qualitative research study in Tanzania and Sri Lanka reported that health practitioners in both countries regularly experience power outages and unstable internet connections that limit the ability to view or upload patient data and the option of using the internet to support clinical decision-making, as well as making data entry more time-consuming [51].

Fragmented care across healthcare providers and between levels of care was perceived as a contributing factor that mitigates the care coordination, influencing the integrated care capacity. As reported in previous studies, insufficient coordination and institutional fragmentation, exemplified by weak coordination between the Ministry of Cooperatives, Labor, and Social Welfare and the MOHME, as well as health insurance plans, were noted as key challenges [52–54]. Further study on the experience of Iran’s health system in implementing e-referral identified a significant gap in interdepartmental coordination, which affects the proper functioning and performance of healthcare referrals [55]. Similarly, evidence from other LMICs indicates that poor integration both across health sectors and between healthcare facilities remains an issue. In the PHC system of China, a study revealed inadequate collaboration among governmental bodies and between PHC and hospital settings, which hinders the continuity of care [56]. Similarly, a qualitative study in rural Guatemala reported that 97% of participants experienced fragmented care across health sectors and 90% of patients sought multiple care providers, further highlighting substantially fragmented diabetes care and limited continuous care [57].

Our findings identified accessible healthcare services as another overarching theme, comprising affordability and coverage, and medications and supplies availability, along with their facilitators and barriers. Our analysis indicates that the expansion of basic health insurance coverage, the availability of essential medications covered by insurance schemes, such as SSO insurance, and the supply of essential medical equipment in PHC facilities served as facilitators, improving availability, affordability and care coverage. However, pharmaceutical expenses for diabetes, particularly at complicated stages, remain high in some settings. One study emphasized that although the population was insured through SSO insurance, significant out-of-pocket expenses for medical care persisted among many households in Iran [58]. In line with this, a study found that the addition of insulin to therapeutic schedules increases the risk of catastrophic pharmaceutical expenditures, especially in the absence of financial protection. Poor households and patients who are resistant to first-line treatments face a greater financial burden in Iran [59]. A systematic review of diabetes treatment costs in LMICs also indicated that diabetes imposes a significant financial burden, disproportionately affecting vulnerable households [60].

Another theme that emerged from our analysis was team-based healthcare services, encompassing healthcare workers’ availability, job demand, and responsibilities of the care team as well as empowerment and competency of the care team. Although task distribution and collaboration through the SIB and regular training of health providers organized by the medical university promoted a positive experience, several barriers, such as a shortage of trained health workers in diabetes care, task diversification and workload, non-clinical task overload, insufficient motivation due to high workload, inadequate knowledge in managing diabetes care, and lack of confidence undermine the effective team-based care capacity.

Shortage of trained health workers for NCDs, particularly for diabetes, within PHC facilities may be addressed through the training of healthcare workers and task management and distributions by the SIB, which equips healthcare workers to provide effective healthcare services. A study highlighted the importance of trained community healthcare workers in managing NCDs such as cardiovascular disease [48]. Moreover, qualitative studies in Cambodia [61], China [56], India [62], and Uganda [63] also reported shortages of skilled healthcare workers and recommended targeted training of healthcare workers for diabetes care. The study in Cambodia [61] also noted that although diabetes management was included in medical and nursing students’ curricula, general practitioners and nurses often lack confidence in treating diabetes patients, particularly those with complications, and expressed a need for specialized training. Similarly, findings from a study in Tanzania revealed that health providers, despite having some knowledge of their expected roles in diabetes care, felt inadequate and ill-prepared for diabetes care [64], an issue that was also reflected in our findings.

In addition, task diversification contributes to a high workload and, as a result, negatively affects healthcare workers’ performance in providing effective diabetes care in PHC facilities, despite the availability of regular training programs. A study conducted in Iran outlined that the inadequate number of healthcare workers for diabetes care, such as dietitians, nurses with expertise in diabetes, and diabetes physicians, poses some challenges, leading to increased overload of tasks [65]. This is also supported by a qualitative study among physicians in Odisha, India, that identified multiple responsibilities of physicians and overburdening as key barriers to the quality of care [62]. Therefore, the recruitment of trained and specialized healthcare workers in managing diabetes care in PHC facilities, as suggested by participants, may serve as a potential fundamental solution. In line with this, a study highlighted that providing training workshops to non-physician community health workers can build knowledge and skills related to NCD care; however, such interventions cannot replace comprehensive formal academic training or hiring more trained and skilled health workers [66]. We illustrated these causal effects through the “shifting the burden” archetype, in which short-term, quick-fix solutions are favored over addressing the root causes of a problem. Although symptomatic quick-fix solutions offer a rapid response, they may impede the ability to allocate time and energy to the fundamental solution. This dynamic increases the reliance on quick fixes, obstructing comprehensive long-term problem solutions [43, 45].

Moreover, this study reveals the continuous healthcare services capacity as an underlying theme of our analysis. Adherence to treatment and self-management support and education, together with their corresponding facilitators and barriers, were grouped into the continuous healthcare services capacity. These findings underscore that the provision of a comprehensive continuum of care by healthcare workers guided by IraPEN guidelines and the SIB enhanced patient awareness, literacy, empowerment, and strengthened treatment adherence, particularly within the rural family medicine program. A study found that the family medicine program in Iran contributed to a significant improvement in diabetes screening and awareness; nevertheless, this program had no impact on diabetes prevalence and control [67]. However, our findings highlight that time constraints in delivering effective case management, weak infrastructure for education and self-management support, and patients’ unwillingness to change behavior and engagement are underlying factors limiting continuous care. We captured these root causes through a “limits to growth” archetype in which the dynamic interactions of a reinforcing loop and a balancing loop are illustrated. A reinforcing loop indicates that increased effort improves performance, promoting additional effort and growth. As the system’s growth continues, a balancing loop driven by an external resource constraint or a limiting condition reduces performance [43, 45].

Furthermore, findings from other studies are consistent with the constraints identified in our research. A study identified inadequate knowledge of diabetes and its complications, patients’ reluctance and unwillingness to engage with care, and limited awareness of public healthcare services for diabetes present major hurdles [65]. A study in rural Uganda similarly noted that limited time, a lack of clinical and educational guidelines, and challenges in providing lifestyle education due to patients’ habits hinder effective patient education [68]. Moreover, a qualitative study in South Africa revealed that patients were not adequately equipped to take an active and empowered role in self-management in this setting [69].

### Strengths and limitations

This study has several limitations. First, despite interviewing a range of health workers, including health officials, urban health technicians, rural and urban GPs, and rural CHWs, we were not able to interview other PHC team members, such as nutritionists and mental health technicians, because of their unavailability in the selected health facilities or their lack of interest in participation. We also conducted interviews only with patients within the primary care facilities who were attending or willing to visit the facility by the invitation of the healthcare workers; therefore, we did not capture other patients’ drivers outside of the PHC facilities, such as individual, cultural, and socioeconomic determinants, which may influence the (in)effective coverage. Although this study was implemented in a district in Isfahan, the healthcare services structure of the district was similar to that of other districts of the province through a PHC network. The strength of the study lies in recruiting a range of participants from urban health centers, health posts, rural health centers, and village health houses. We also combined rigorous qualitative methods with a systems thinking approach and systems archetypes.

### Implications

The findings of this qualitative study, using a systems thinking lens, highlight root causes influencing effective coverage of diabetes care in PHC in Iran. Health workers drew attention to weak coordination across various providers and levels of care, deficiencies in the electronic health information system, and inadequate numbers and limited capacity of healthcare workers for diabetes care in the PHC. Both health workers and patients emphasized the financial burden associated with diabetes with complications, particularly given that not all medications are included under health insurance. Both groups also outlined time constraints among healthcare workers that hinder effective communication, while healthcare workers further underscored the importance of patient engagement throughout the care management. In practice, these findings suggest that health policy makers should prioritize strengthening digital health infrastructure, improving care integration both across various health providers and between levels of care, enhancing financial protection for patients, particularly individuals who are resistant to first-line treatments or who are experiencing diabetes with complications, expanding the recruitment of trained and skilled healthcare workers for diabetes care, and promoting patient engagement. Moreover, systems thinking through robust tools such as a causal loop diagram and systems archetypes can assist health decision makers in better understanding the root causes of suboptimal effective diabetes care coverage to target key leverage points for successful strategies.

## Conclusion

In summary, this qualitative study underscores that achieving effective coverage of diabetes care requires ensuring integrated, accessible, team-based, and comprehensive continuous care. Efforts such as integrating diabetes care into the PHC context, expanding basic health insurance coverage, and implementing standard clinical guidelines and electronic health information systems positively contribute to diabetes management; however, persistent constraints such as fragmented service delivery, limited accessibility, weak team-based care, and poor continuous care negatively affect the effective coverage of diabetes care in Iran.

## Electronic Supplementary Material

Below is the link to the electronic supplementary material.

**Supplementary material 1: **Appendix 1

## Data Availability

The datasets generated and/or analyzed during the current study are not publicly available due to confidentiality obligations. The participants did not provide consent for their interviews to be shared or analyzed beyond the scope of the present study. De-identified portions of data are available from the corresponding author upon reasonable request.

## Abbreviations

NCDs: Noncommunicable diseases
PHC: Primary Health Care
MOHME: Ministry of Health and Medical Education
SSO: Social Security Organization
IHIO: Iran Health Insurance Organization
