# Supplementary material for: Exploring underlying factors associated with effective coverage of diabetes care in Iran: a systems thinking approach
Source: BMC Health Serv Res. 2026 Jul 24;26:1436. doi: 10.1186/s12913-026-15160-3 (PMC13682105; doi:10.1186/s12913-026-15160-3)
Supplement: Supplementary file 1 [file 12913_2026_15160_MOESM1_ESM.docx]

**Interview guides**

**Introduction**

I am [interviewer name, position, and affiliation]. I am conducting the current research to understand factors influencing effective coverage of diabetes care in Isfahan province. I am interested in inviting you as one of the participants in this study. Please ask about any words/ concepts that you do not understand. You do not have to answer any questions if talking about them makes you uncomfortable.

I would be grateful if you could participate in this study.

Do you consent to participating in the interview in accordance with the informed consent?

Yes  No 

Date of interview: …

Place: …

Do you consent to the audio-recording of the interview for the ease of transcription and analysis?

Yes  No 

1. **Demographic Information of Health Workers**
   1.1. Gender: Man	Woman	Other
   1.2. Education: High school diploma/associate degree  Bachelor’s degree Master’s degree Doctorate 
   1.3. Age: ….
   1.4. Professional qualification and your role: …
   1.5. Work experience: …
   1.6. Area of service: Rural , Urban
2. **Interview questions for health workers**
   2.1. How, where, and by whom are diabetic patients screened, diagnosed, and treated?
   2.2. How are services for diabetes provided during screening, diagnosis, treatment, and control?
   2.3. Can you explain any barriers (or delays) along the patient pathway in each step of the cascade of care (screening, diagnosis, treatment, and control)?
   2.4. What factors would be the most influential in improving the healthcare services for diabetes? Why?
   2.5. What factors contribute to the loss of diabetes patient cases at the stages of the cascade of care? Why?
3. **Demographic Information of Patients**
   3.1. Medical condition: Type 2 diabetes , Type 2 diabetes with other comorbid such as hypertension, cardiovascular disease, dyslipidemia, etc. 
   3.2. Gender: Man	Woman	Other
   3.3. Age: ….
   3.4. Education: Below high school diploma  High school diploma/associate degree  Bachelor’s degree Master’s degree Doctorate 
   3.5. Occupation: Self-employed Employee Retired Other
   3.6. For how many years have you had diabetes: …
   3.7. Place of residence: Rural area	Urban area
4. **Interview questions for patients**
   4.1. How, where, and by whom was your disease diagnosed and treated?
   4.2. Since your diagnosis, where and how have you been receiving regular follow-up and treatment?
   4.3. How are diabetes services provided during screening, diagnosis, treatment, and control? How do you describe your experience with the delivery of healthcare services?
   4.4. What challenges and difficulties, if any, have you faced when receiving healthcare services for your disease?
   4.5. Which factors enable you to receive healthcare services for your disease?
   4.6. What factors would be the most influential in improving the healthcare services for diabetes? Why?
